# Complete Automation of an Energy Consumption Reduction Strategy from a Water Treatment and Distribution Facility, Inside an Industrial Internet of Things-Compliant Proactive Historian Application

**DOI:** 10.3390/s21072569

**Published:** 2021-04-06

**Authors:** Andrei Nicolae, Adrian Korodi, Ioan Silea

**Affiliations:** Department of Automation and Applied Informatics, Faculty of Automation and Computers, University Politehnica Timișoara, 300223 Timișoara, Romania; andy_nicolae@yahoo.com (A.N.); ioan.silea@upt.ro (I.S.)

**Keywords:** energy consumption reduction, Industrial Internet of Things, Industry 4.0, proactive historian, water industry

## Abstract

The Industrial Internet of Things and Industry 4.0 paradigms are steering the industrial landscape towards better connected entities, superior interoperability and information exchange, which lays the basis for developing more intelligent solutions that are already starting to bring numerous benefits. The current research aligns to this course, in an attempt to build an automated and autonomous software tool, capable of reducing the energy consumption of a water treatment and distribution facility, by optimizing the water sources usage. Based on several previous researches, the present paper details both the complete automation of the optimizing strategy inside a proactive historian application and the tests executed with the finished solution. Possessing the abilities to directly influence the monitored system in a non-invasive manner, and to link all the sequences of the algorithm automatically, the solution is now ready for long-term functioning without any external interference.

## 1. Introduction

As a first remark, the primary point of interest regarding future research and development in the contemporary industrial environment gravitates around improvements in availability, safety, productivity and cost reduction, all of them obtainable through better interoperability, connectivity and information exchange between different industrial entities. Certainly, those new links between previously isolated mechanical systems would set the required conditions for the emergence of intelligent software solutions that, eventually, will be capable of optimizing technical systems and maximizing their performances, with directly measurable benefits for all parties involved. This relatively recent, wide spectrum, fast advancing, high potential, huge interest drawing research direction from the industrial branch is guided at the conceptual level by the Industrial Internet of Things (IIoT) [1,2,3,4,5] and Industry 4.0 [6,7,8,9,10] paradigms, both very similar approaches which are pleading towards the introduction of digitalization into industry and connecting the physical world to the Internet [11]. The potential benefits of this endeavor are not neglectable in any way and will be better highlighted with the introduction of more innovative technological solutions, such as the ones described in [12,13,14,15,16,17,18,19,20,21,22,23].

Narrowing the focus on the water industry, which represents the deployment target of the current research, the specific landscape of this domain is still predominated by legacy systems, consisting of a large variety of technical solutions and processes, contributing to a heterogeneous, chronologically dispersed perspective and even inefficiency, if modern standards are considered. As a consequence, this field can definitely provide room to receive significant enhancements. The general perspective in the manufacturing industry is more or less the same.

Nevertheless, the aforementioned IIoT and Industry 4.0 ideas have gradually begun to make their presence felt in the water industry as well, initially tackling the interoperability issue with the introduction of a standardized, platform independent communication architecture, the Open Platform Communications Unified Architecture (OPC UA) [24,25,26,27] and various advances in the networking area, such as time-sensitive networking (TSN) [28] or the publisher-subscriber model [29], among others. After this initial development mostly targeted infrastructure-level services, other relevant researches began to emerge in this context, even towards the energy consumption of this industrial branch, similar to the ones from [30,31,32]. 

Enjoying this kind of progress in place, the data accumulation phenomenon is already encountered in real world industrial environments, being also spurred in recent years by the increased availability of low-cost, lightweight, open communication protocols compliant, open data access historian type of software applications. On this line, the development from [33] was specifically tested in the water industry, while the studies [34,35,36] offer alternatives for other industrial areas.

After gathering large quantities of data, using a historian application, the next natural step is to determine a way to use this data, by analyzing it, identifying patterns, setting objectives and ultimately develop proactive tools, capable of optimizing the monitored technical system, in an autonomous regime, without human intervention or supervision. The purpose of investing in this step is represented by increases in performance, efficiency, productivity and/or decreases in operational costs, pollution in many industrial activities. The task of obtaining such tools, at a technological readiness level that would allow them to run autonomously, without constant human supervision, on critical infrastructure, such as the water infrastructure, requires a tremendous research effort, sustained over a long period of time. However, small, incremental progress on this path is currently being reported, for example, the study from [37] is attempting to use historian gathered data from the water industry for predictive control of a water distribution network. In the same direction, article [38] introduced a 3-level reference architecture for a proactive historian, presenting the software elements that need to be added to an elementary historian in order to elevate it to the proactive level. Besides the theoretical development, the article contribution expanded towards the practical area, by implementing the first level of the respective architecture and testing it on a drinking water treatment plant (DWTP). Then, the research from [39] improved the solution from [38] and implemented a weather-based prediction strategy, as part of the second level of the reference architecture, applied on a wastewater treatment plant (WWTP). Continuing on this already well established research topic, article [40] brought a leap forward with its research of an energy consumption reduction strategy inside a DWTP, based on prioritization of the water sources. The tests executed on real world processes delivered the remarkable result of 30% energy consumption reduction, without any output water quality or equipment maintenance alteration.

Considering the developments and results achieved to date, the current research paper contributes to the effort towards obtaining a proactive historian able to optimize the supervised industrial system. More precisely, this study attempts to continue the strategy developed in [40] and to integrate completely the strategy into a proactive historian software application, in order to obtain the capability of influencing a DWTP’s water sources functioning, in order to reduce the energy consumption, in a non-invasive, autonomous, unassisted operation. The paper approaches mainly the automatic identification and adaptation of a water well quality indicators throughout continuous automatic long-term analysis inside the proactive historian, but also further detailing, adjustments and testing of implementations and concepts within the proactive historian in order to ascend technological readiness level scale. 

In the context of a functional industrial system, with an already implemented automation of the technological process, the local system will operate according to its algorithm an its imposed limits throughout its entire lifecycle. Invasive interventions at the programmable logic controller (PLC) or supervisory control and data acquisition (SCADA) levels to implement changes are always avoided due to many reasons. Some basic and very important reasons would be: the difficulties in the actual intervention caused by lack of source codes/projects, lack of documentations, the complexity and implications of a basic alteration in the core system, the general legacy nature of the approached structures (e.g., outdated hardware and software equipment, lack of software development and programming environments, lack of development licenses, lack of firmware upgrade possibilities for PLCs, lack of available licensed additional SCADA modules to implement the desired algorithm), the requirement to not stop the system (e.g., a PLC intervention generally causes a stop of the system; a simple start/stop procedure in the industry on a legacy or not so well developed SCADA application can frequently cause 4–5 h of downtime), etc. Also, the problematic interference in a functional industrial process may be limited by various warranties, maintenance contracts, that are not meant to improve algorithms, but to manage existing fix functioning. When talking about critical infrastructures (e.g., water, electricity, gas, etc.) or time-sensitive manufacturing plants, the invasive interference is excluded until the next upgrade of the whole local system, and in automation, this type of upgrade is realized after very long functioning times, causing massive legacy equipment being present in any industry. Going further, the industry lacks distributed data accumulation structures, and when present, the historians are mostly unused components or used in a very limited manner because of their lack of proactivity, lack of process awareness, the incapacity to identify data dependencies, to determine efficiency increasing strategies and to react over the functional systems. Even if a historian is present, the accumulated data is waiting for the human eye to maybe find a dependency or a recipe in a long-term behavior of the system, and obviously the amount of data is not possible to be handled in this manner (e.g., one small DWTP can contain around 2000 tags without considering audit-based tags, meaning 2000 values/sampling times, with in larger plants there can be as many as 8000 tags). The process functioning can be altered over time (physical process and equipment degradation, operator’s behavior change), and the data has to be analyzed permanently. Following data dependency analysis, the historian must elaborate efficiency related solutions based on objective functions and constraints, in a process aware context. Also, the historian has to be able to react over the local system according to the identified efficiency improvement strategy, in a non-invasive manner (e.g., change references in a local control loop considering all the mentioned constraints), to commission the elaborated strategy. A completely automated solution means that the distributed historian is able to do all the above-mentioned actions throughout the functioning of the local system, autonomously and automatically, in a process-aware context, without any human intervention. The mentioned complete automated historian based solution was not encountered in the literature or the industry. 

Therefore, the current paper is highly interconnected to prior researches undertaken by the authors and builds on top of previous developments, its main contributions being mainly directed towards filling the gaps that prevented the fully long-term automatic functioning of the strategy researched in [40], which, besides the scientific target, would outline the practical creation of a proactive, intelligent, IIoT-compliant tool, drawing considerable interest from the water industry companies.

The general motivation for pursuing the above goals presents a dual characteristic: (i) reducing the energy consumption of a DWTP leads to reducing the operational costs, which should, theoretically, reflect in the population’s water bill decreases, which, in some parts of the globe, could mean improved accessibility to drinking water; (ii) depending on the methods used to generate the consumed energy, a reduction could mean a fossil fuels consumption reduction, which has a positive environmental impact.

Section 2 addresses topics related to DWTP particularities which have an impact on the current research, presents previous works whose results are useful in this case and details the researched and implemented solution which represents this paper’s contribution. Section 3 reveals the results obtained while testing the solution, results and findings which are further discussed in the Section 4. Section 5 presents the conclusion.

## 2. Materials and Methods

### 2.1. Relevant DWTP-Specific Topics

This section presents a limited-details perspective, containing only a summary of those parts and topics from a typical DWTP process that plays a role in the current research. On the other hand, a more detailed and complete image over the processes that take place inside a DWTP can be formed by consulting either [38] or [40].

**Figure 1:**
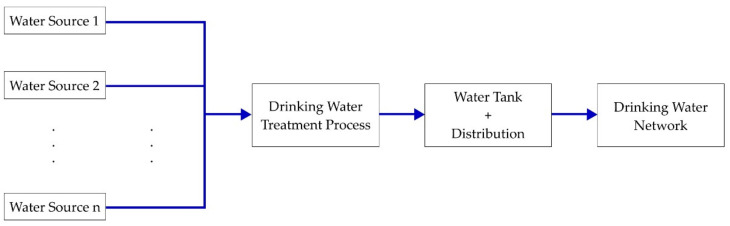
Simplified overview of the stages taken by the drinking water before entering the consumer network.

First, a simplified representation of the water journey, starting from natural sources and continuing until the moment of entering the drinking water network, is illustrated in Figure 1, below, and discussed further in the sections that follow.

Taking into account the above schematization, it can be observed that a typical DWTP relies on multiple distinct water sources, the most common type of source being, at least in the authors’ geographical area, drill holes in the ground, from which water is extracted, with pumps, from the ground water table. The water from all the sources is mixed into one total flow, which is delivered at the treatment process input. The treatment process cleans the water and ensures that, at its output, the water quality meets the legal drinking water requirements. After treatment, the resulting water is placed inside water tanks, and sometimes additionally a water tower, from which it is distributed into the drinking water network. The water tanks acts, more or less, like a buffer between the network, which is extracting water from it, and, on the other hand, the treatment process, which adds water to it. The water level inside this tank dictates how much water the sources should provide. However, the total mixed flow, directed at the treatment process input, in which all running sources introduce water, is the one requested by the plant’s automation, based on water level inside the tank and by the actual output flow low within the distribution network, which leads to the ascertainment that both the way this total flow is split between sources and the usage of sources are decided in practice only regarding the functioning hours of water sources.

Regarding the energy consumption, it represents the consequence of industrial equipment functioning inside the DWTP, large consumers being water pumps and air blowers, the latter being used in the treatment process. In this context, an obvious observation is that equipment functioning time reduction leads to lower energy consumptions. However, a reduction in equipment functioning means a reduction in the amount of treatment applied to the water, which, in order to maintain the DWTP output water quality, implies a better water quality at DWTP input, the logical reasoning is that the better the water quality is at the DWTP input, the less treatment it will require, which means less energy consumption.

In terms of water quality, in practice, there are noticeable differences between the water sources of a DWTP. As a small parenthesis in the discussion, also of interest for the current research is the difference of flow capacity that each source can offer. Returning to the water quality, when a new water source (well or surface water) is put into service, a technical datasheet is being made, where different parameters of the water are written. Those parameters are identified by taking water samples from the source and sending it to a laboratory for analysis. It is important to underline that there is not single parameter that perfectly reflects the quality of the water, but rather a series of parameters which can be considered, together, although a generally accepted formula for computing a universally valid quality indicator from the group of quality-indicating parameters does not exist. In the real world, the problem is even more complex, because a source’s water quality is always changing over time as a result of different factors, such as pollution or overuse. Despite this change, in practice, the laboratory analysis is not periodically repeated after the initial entry into service, partly because of the test costs and partly because of the trouble of taking samples, so, the result, backed up by studies on around 50 DWTPs made by the article authors in recent years, is that the quality indicators of the water from sources have not been taken into consideration until now, at any of the implemented automation solutions. The best case scenario encountered in practice is when local plant operators have noticed, over time, that if they request more water from specific sources, then the plant equipment seems to work less than if they request water from other sources, but those are empirical and subjective conclusions, established by chance observations, not by scientific methods.

The most important conclusion from this section, that needs to be emphasized, is that the energy consumption of a DWTP is directly influenced by the quality of the water introduced at its input. Furthermore, this water quality can be adjusted, within certain limits, because it is composed from a mix of the quality of each source that is delivering water in that specific moment, depending on their flows, as indicated in the following equation:

Q_t_ = Q_1_ × W_1_ + Q_2_ × W_2_ + … + Q_n_ × W_n_ (1)

where Q_t_ represents the quality of the water introduced at the DWTP input, Q_1_ … Q_n_ represent the quality of the water from sources 1 … n, while W_1_ … W_n_ represent the weighting factors of each source, these factors being equal to the percentage that the current flow from the respective source (how much water is currently being delivered by the respective source) represents in the total flow delivered at DWTP input. By adjusting the current flow of individual sources, the weighting factors are directly influenced, and, thus, can be used to improve the quality of the water introduced at DWTP input (Q_t_).

The fixed, rigid requirement, imposed in real time by the DWTP automation, is the total flow (quantity) of water that must enter the DWTP, namely Q_t_. The decision regarding the way this quantity is reached, in essence, how much water is requested from which sources, is taken arbitrarily at individual DWTP level. It is worth mentioning that, for the same total quantity of water, different water quality can be obtained at DWTP input, depending on which sources are used and what quantity each source provides, but the real world setting shows that, although both the quantity of water than can be delivered (minimum and maximum limits) and the quality of water differs for each individual source, the sources are not being used in a way which follows the clear purpose of obtaining the best quality for the water at the input of the DWTP. This reality means that all the encountered DWTPs are currently running above their optimum energy consumption.

### 2.2. Related Research

As previously stated, the research presented in this paper is highly interconnected with other studies, developments and researches made by the authors in recent years, which are indispensable for allowing the current exploration to proceed. Because of this fact, the present section reviews this previous work and offers brief explanations regarding the reasons why it is necessary to refer it in the given situation, in order to facilitate a better understanding of the context in which the current research is placed.

Any optimization attempt must rely on solid data, recorded from the monitored technical system, such that it is imperatively needed that the primarily used tool is going to be a historian type of software application. The answer to this was provided by [33], which proposed a software application entitled Historian, which, to the difference of other available solutions, is lightweight, can be installed on a compact Raspberry Pi hardware platform, uses the open OPC UA protocol and does not hide the stored data under proprietary data access protocols. Furthermore, the practical implementation has been tested exactly in the water industry, on real world equipment, making it suitable for the current study.

Another crucial contribution that is used in this research is the data dependencies identification module, developed and integrated in the Historian from [33] during the research [38]. In order to reduce the energy consumption, there must be a method of identifying how the technical system characteristics (water flows, functioning hours, substances consumptions, energy consumptions, equipment faults, etc.) influence and depend on each other. The algorithm from [38] and its practical implementation inside the Historian (started in [38] and improved in [39]) perfectly meets this demand, by offering the information, when the measured values, over time, of two characteristics are available, if those values evolve, in time, proportionally or not regarding to each other and to what extent.

When recording the measured values of different characteristics of a system, any historian type of application receives, from the system, a series of numerical values associated to a tag, which is a string of characters (tag examples: D1_max, OPCTpH1UA, CSF9_Frecventa, etc.). Obtaining a complete automation of an energy consumption reduction strategy, which does not require human assistance, assumes that the automation tool understands the practical significance behind those strings of characters. The automation tool must understand, for example, that the tag ‘F3_Debit_T’ signifies the water flow that is currently being offered by the water source with number 3, and the role of the structure in the whole process. Practically, the idea is to have a process-aware historian, an idea that was not found in literature or practice. The problem of attributing a specific meaning, inside the Historian, to the tags that are being monitored was tackled in [39] and solved with the introduction of the so-called “Historian Process Editor”.

Considering this article’s optimizing objective, the most important previous work is described in [40], where the research team developed, at conceptual level, a complete strategy for reducing a DWTP’s energy consumption, by prioritizing its water sources and determining the flow reference for each local control loop. Furthermore, the research from [40] was applied in the practical sphere as well, sustained by a MatLab Simulink simulation, where the strategy was tested with input data from a real DWTP, before being tested on real systems, the latter with limiting constrains imposed by the local water company. Despite the fact that the practical approaches from [40] proved that the strategy is efficient in reducing energy consumption, some aspects require more research to obtain a completely automated long-term functioning of the proactive historian associated to the real system. During the tests executed in [40], the water sources priority indicators were determined by corroborating the experience-based input from local operators with various dependencies identified by the Historian data analysis module which was implemented in [38]. However, the procedure required a person setting different time intervals for the analysis, in the Historian graphical user interface (GUI), executing the dependencies analysis repeatedly and noting any relevant outcomes. Also, because the water company did not allow changes at water sources requested flow set-points during tests, the practical implementation of the theoretical strategy which divides the total flow requested from all sources into specific flows for each source was not applied in the real scenario. The resulting software component followed the general theoretical blueprint, but it was truncated in real testing and did not cover all possible scenarios and corner cases. Also, the alteration of the system operation in the context of the strategy response actions over the real system, meaning the starting and stopping of water pumps at the water wells and miscellaneous permitted minor adjustments were highly monitored and limited to human assistance. Although this approach worked and delivered very good results in the test scenarios from [40], for a large scale deployment and long-time autonomous functioning of the solution in the water industry field, more research is needed. A completely automated approach will assure that this strategy can bring substantial benefits in the real world, outside research trials circumstances.

### 2.3. The Implemented Solution

Having at the disposal of the authors the developments described in the previous section, only the following gaps are currently preventing the complete automation of the energy consumption strategy detailed in [40]:

- Lack of a completely automated method, without introducing additional overhead, to determine the water quality indicator of each water source (periodic computation of *q_f_* from Equation (3) in [40]);
- Lack of a robust, with all possible cases covered, practical software implementation of both the series of formulas from [40] and the algorithm for dividing the necessary flow between sources, introduced in the same article. This software should be capable of directly applying the conclusions, in a non-invasive manner, inside the monitored system.

The abovementioned gaps represent this paper’s concern and the fixes to those problems represent this paper’s main scientific and practical contributions. At this point, the very complex context in which this paper is set has been brought to consciousness, which means the rest of the current section will focus on this paper’s contributions and implemented solutions.

From the start, it is very important to understand that any DWTP which wants to benefit from this energy consumption reduction tool’s perks must meet a series of prerequisites:

- The DWTP must have at least two water sources;
- The following data must be provided from the technical system to the optimizing tool, for a period of time of at least 2–3 months before any optimization can be applied: current water flow for each source (actual), reference water flow for each source (set-point), minimum possible water flow for each source, maximum water flow for each source, an indicator regarding how much each source has been used until now (e.g., functioning hours of water pumps, number of starts), total flow requested from all sources, total DWTP energy consumption. This data will be used for analysis, in order to identify patterns and dependencies;
- For each data described at the second bullet, an operator must associate a meaning, inside the optimizing tool, to the respective tag (the optimizing tool must precisely know, for example, if the numerical values of tag X represent the total water flow or the energy consumption);
- The automation that is already present inside the DWTP, ensuring its normal operation, and it must have control loops implemented in such a way that they use, as a reference for the water flow that each source should deliver (set-point) the values attributed to an OPC UA tag. This condition allows the optimizing tool to influence the system in a non-invasive manner, without requiring any changes to the DWTP automation’s control loops. This way, the tool changes tag values in order to alter the system’s functioning.

Considering, on the one hand, the fact that all of the indispensable previous work has been implemented in the proactive Historian software platform and, on the other hand, the need to impose the abovementioned prerequisites, it was decided that the best approach for obtaining the desired complete automation of the energy consumption reduction strategy would be to integrate the practical development inside the Historian, targeting an accordance with the level 3 of the software architecture described in [38]. The starting basis has been taken at the Historian development point presented in [39], the current paper building on top of the respective level.

Ensuring that all the required data is available and the prerequisites are met is done with the help of the Historian Process Editor that was introduced in [39], and that induces the process-aware historian concept. More specifically, if the optimization strategy is used, it was enforced that the currently used process has an OPC UA tag, from the DWTP’s system, attributed to all of the characteristics mentioned in the second bullet of the prerequisites. Also, a convention was set that the object ‘Flowmeter 1’ indicates the total flow requested from all sources and the object ‘Energy Sensor 1’ indicates the DWTP’s total energy consumption, while the presence of those objects, along with at least two water sources is also enforced. 

**Figure 2:**
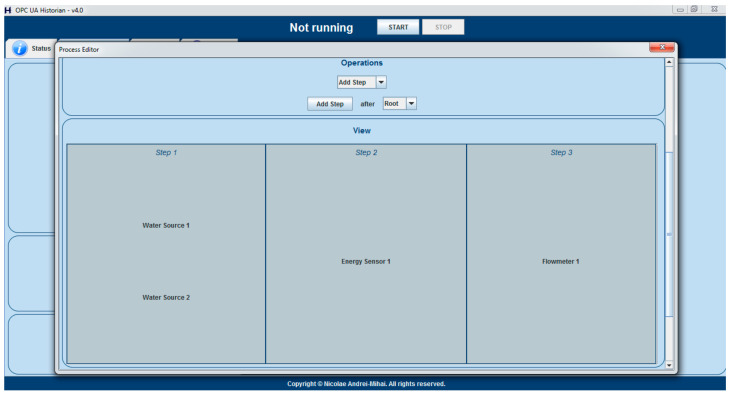
Example of the minimum objects that must be defined, inside the Historian Process Editor, for the currently used process, in order to execute the optimizing strategy.

**Figure 3:**
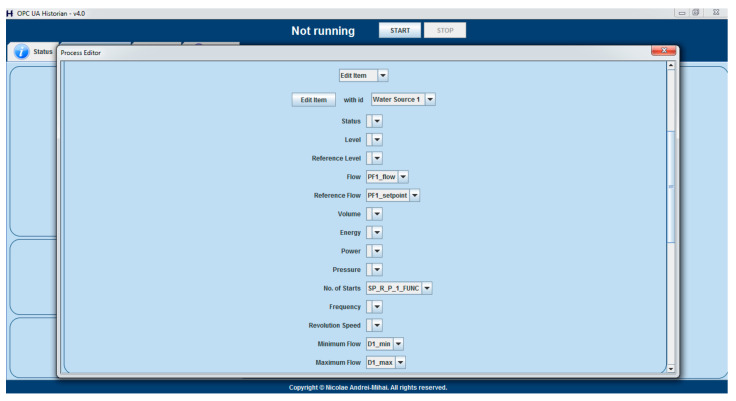
Water source characteristics that must have tags associated, inside the Historian Process Editor, for the currently used process, in order to execute the optimizing strategy.

As an example, Figure 2 above illustrates the minimum components that the currently used process should be made up from, while Figure 3 below presents the minimum characteristics of a water source that must have their tags associated for the optimizing strategy to run.

**Figure 4:**
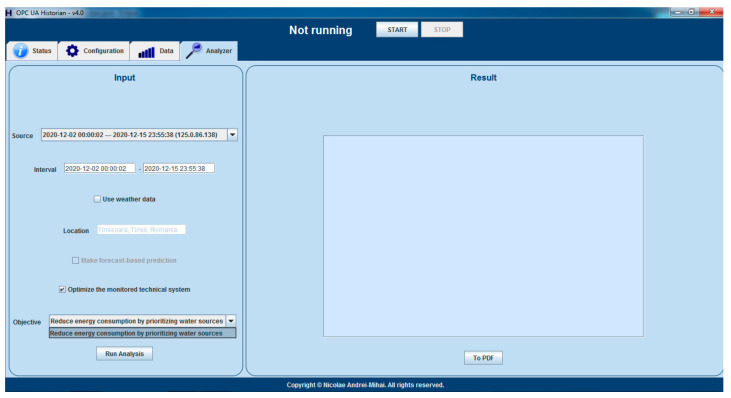
Optimizing objectives choice inside the proactive Historian application.

By having all the input data available for the optimizing strategy, the next step is to include the solution in the general, generic proactive architecture, in which the Historian will, at some point in the future, offer multiple optimizing objectives, at level 3 of the reference architecture, from which a DWTP stakeholder will be able to choose. In order to fulfill this long-term concept, the Historian GUI was improved, as presented in Figure 4, for offering the optimization objective choosing possibility, the energy consumption reduction being the only option available to date.

The implementation of the automation of the strategy from [40] began with a conceptual-level identification of a method to compute the water quality indicator of each source (*q_f_* from Equation (3) in [40]), without requiring any human assistance or any complicated and expensive water sampling and analysis. The answer to this problem can be found by capitalizing on the idea that the DWTP overall energy consumption is directly influenced by the water quality at the DWTP input. Adding the fact that a higher flow of water from a specific source means that the quality of water from that source has a bigger influence on the total quality of the water at DWTP input, the authors draw the conclusion that the water quality of a source can be determined by analyzing historical stored data and correlating the water flow changes of that source with DWTP energy consumption changes. This means that the water quality of sources can be determined by analyzing flows and energy consumptions recorded while the DWTP functioned in the past, without executing any laboratory analysis on water or measuring any other quality-specific indicator. Of course, a data analysis tool is required, but the Historian application already has such a dependencies identification algorithm, implemented in [38], which facilitates the implementation of this experimental method, covering the most important aspect that prevents the complete automation of this optimizing strategy.

From the practical standpoint, the execution of the energy consumption reduction strategy requires a previous successful execution of the dependencies identification algorithm from level 1 in the reference architecture because the results of this first level algorithm serves as input for the optimizing strategy execution.

**Figure 5:**
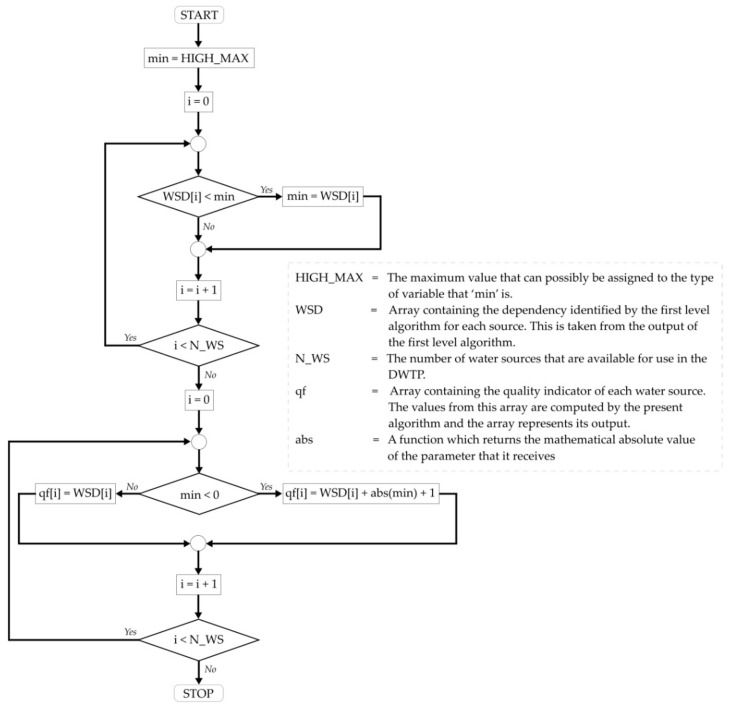
The implemented algorithm which determines *q_f_* from the results of the first level dependencies identification algorithm.

The algorithm which determines *q_f_*, by starting from the results of the first level algorithm, is presented, in the form of a logical diagram, in Figure 5, below, and further detailed after the schematization.

The dependencies of interest, outputted by the first level algorithm, are the ones when the current water flow of a source (actual value) was set as reference in the analysis and the total energy consumption of the DWTP was the analyzed characteristic. Those roles, alongside the meaning of the dependencies values are detailed in [38]. Those dependencies of interest are extracted from the dependencies graph to an array, named WSD in Figure 5. For the current application, the highest water quality is the one from water sources identified as having flow values evolving inversely proportional to the energy consumption, followed by the not proportional ones, the lowest quality being at the directly proportional ones. If multiple sources are identified as having the same proportionality result, then (a) for directly proportional, the best quality is at the lowest number, and (b) for inversely proportional, the best quality is at the largest number. Again, in order to better understand the reasoning from the last two phrases, an understanding of the first level algorithm results meaning is needed, detailed explanations in this regard being available in [38]. For obtaining the *q_f_* values, in accordance with the aforementioned logic, if there are any water sources that are inversely proportional, their dependency value from level 1 algorithm is negative, so in this case, of at least one inversely proportional, the absolute value of the minimum dependency value is added to all *q_f_* values, as shown in the lower part of Figure 5. By doing so, all computed *q_f_* values are always positive and the priority becomes linear with the values, regardless of the proportionality: lowest number is highest quality, highest number is lowest quality. Due to the fact that the obtained values do not respect the highest number equals highest quality assumption, in order to compute the priority indicator based on water quality, the Equation (3) from [40] was transformed into the following equation, which, applied in the current context, is equivalent to the original:

(2) $${PQ}_{f}=10-q_{f}\cdot \frac{10}{max(q1,\ldots ,qn)}$$

After implementing the algorithm described above inside the Historian, the last piece that was missing from the data required for the Equations (1)–(4) from [40] to be applied is now available. The implementation of those equations calculations inside the Historian, in Java, is quite trivial and does not pose any difficulties, so the details can safely be overlooked, as there is no problem in recreating it after reading the article [40].

**Figure 6:**
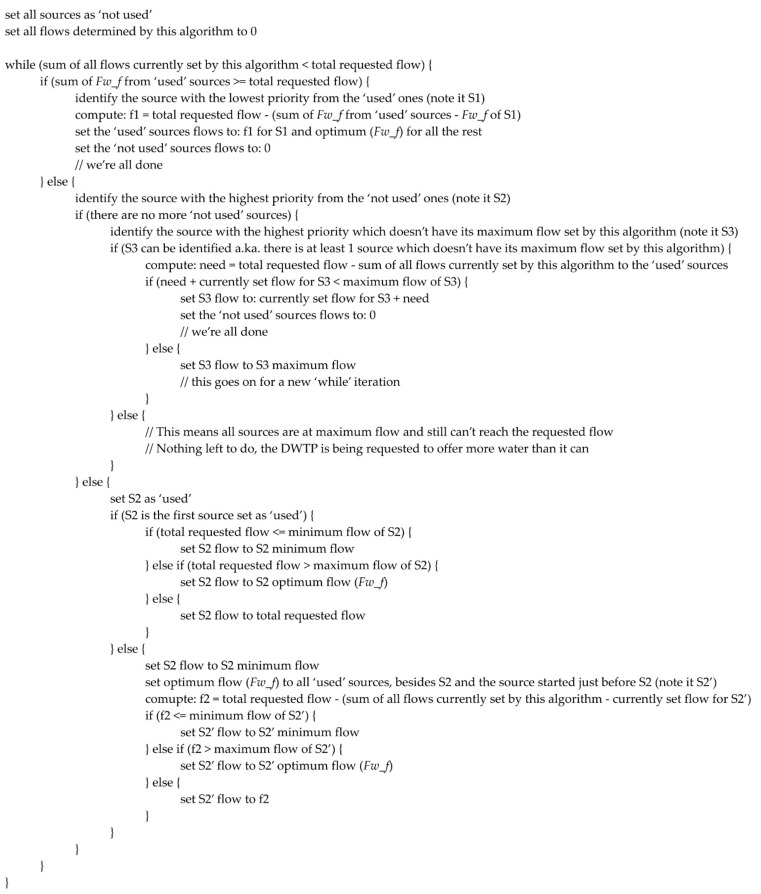
A high-level summary of the implemented algorithm for dividing the total requested water flow into specific flows for each source, in an optimum way for reducing energy consumption.

**Figure 7:**
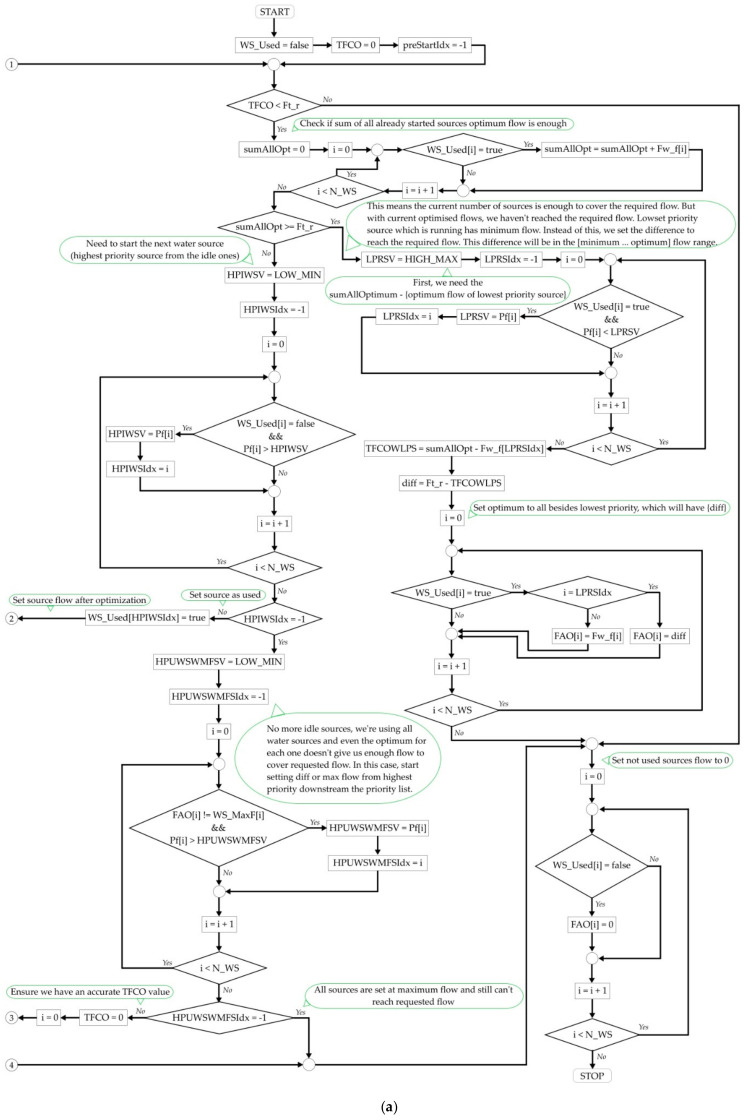

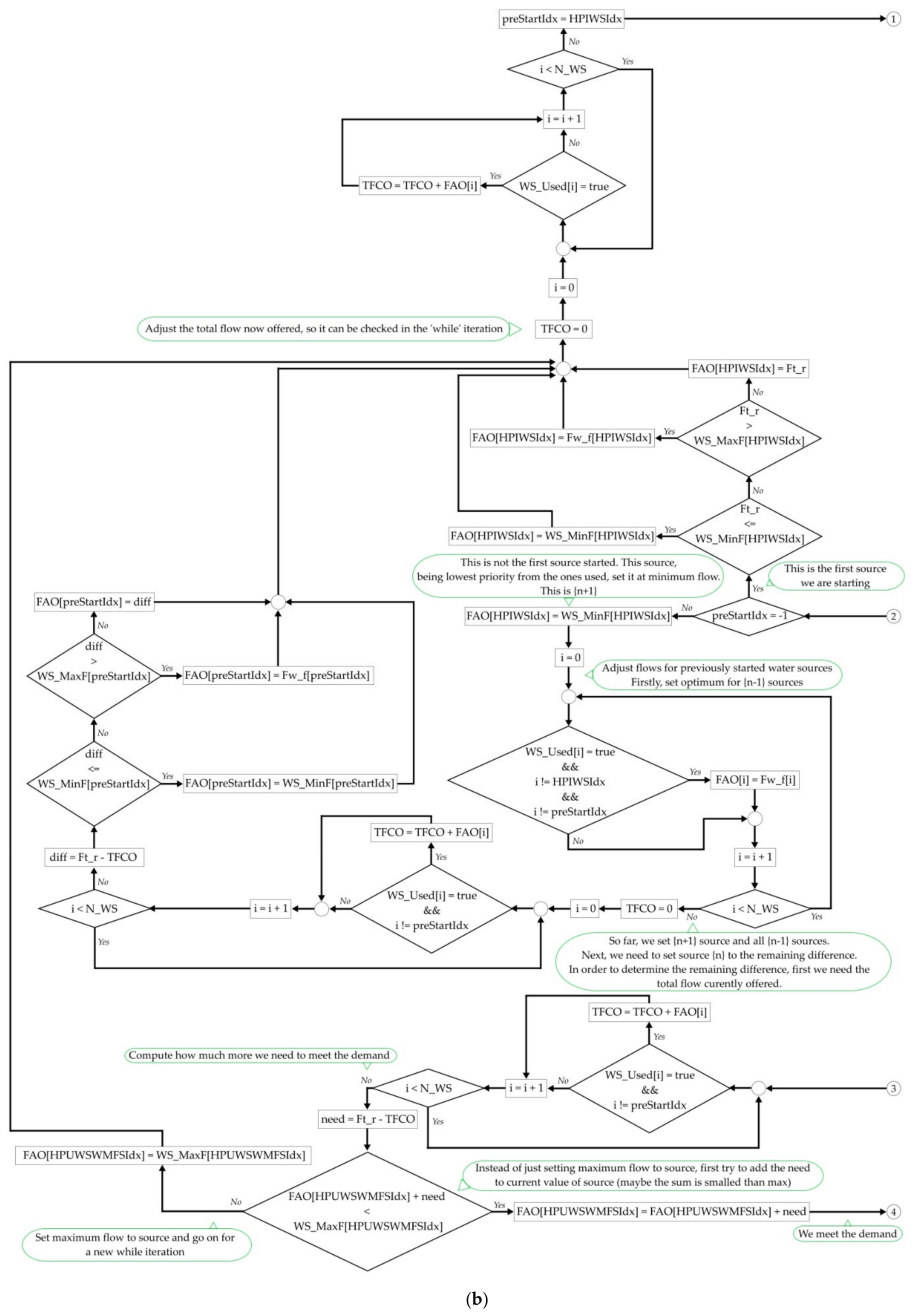
Detailed perspective of the implemented algorithm for dividing the total requested water flow into specific flows for each source, in an optimum way for reducing energy consumption, (**a**) right side of the diagram, (**b**) left side of the diagram.

The last part of the implementation consists of the logic that decides the exact flow each source should offer for an optimum energy consumption. This logic was described, at conceptual level, in [40], but the robust, complete algorithm implemented in this paper, inside the Historian, is described, from a high-level perspective, in Figure 6. However, in order to facilitate possible reproduction attempts, a very detailed perspective was considered, too, the same algorithm following the presentation, in the form of a logical diagram, from Figure 7. 

This figure was augmented with comments, to facilitate the following of the logical thread and an explicative Table 1 was added after the image, to inform regarding the abbreviations from the schematization. Due to the complexity of the algorithm, which could make Figure 7 quite difficult to follow as a whole, this diagram was divided as illustrated in Figure 7a,b, the two parts being logically linked with break connectors. Thus, if only a general, high level idea is expected, then Figure 6 meets this demand, but Figure 7 covers the cases in which a detailed snapshot is required.

After executing the algorithm from Figure 7, the automation tool possesses the optimum flow that should be set for each source (FAO) in order to optimize the energy consumption. Joining this information with the prerequisite of associating an OPC UA tag for the water flow set-point of each source, the problem of finding a method to influence the technical system is also solved.

**Figure 8:**
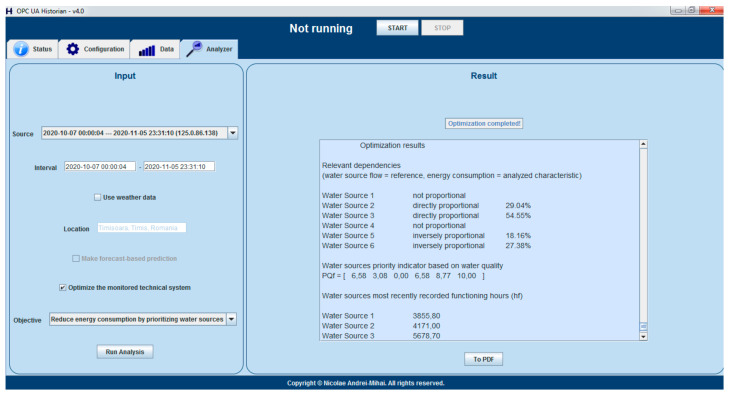
Example of displaying, in the Historian GUI, the results of running the optimization solution.

The step-by-step results of each stage involved in the energy consumption reduction strategy execution, integrated inside the Historian, is displayed on the GUI, as exemplified in Figure 8, allowing external export, in PDF format.

The implementation of this strategy ends with the writing of the computed water flow set-point of each source to the corresponding OPC UA tag used inside the DWTP automation control loops. This way, for the first time, the proactive Historian has closed the loop described in the reference architecture introduced in [38], becoming capable of monitoring a technical system, analyzing the stored data and using the conclusions in order to compute an energy consumption optimization, which is directly applied to the system, whose functioning is influenced by the proactive Historian.

## 3. Results

### 3.1. Step-by-Step Analysis of the Implemented Solution Execution

Maintaining a primordial importance in the testing and validation stages of the implemented solution, verifying the fact that all the computed values are compliant with the equations from [40] represented the initial focus.

In the continuation of this section, a step-by-step presentation of just one optimizing strategy execution, inside the historian, from the collection of performed tests, is exposed, with the intentions of both clarifying the way in which the optimization operates, and detailing the practical result of the implementation. The following sequence of data is extracted directly from the output displayed in the Historian GUI (similar to the one depicted in Figure 8).

As stated before, the optimizing strategy uses the part of the output of the dependencies identification algorithm from the first level of the reference architecture which considered the actual water flow of sources as reference and the DWTP total energy consumption as analyzed characteristic. In this specific case, the output was the one from Table 2.

Using the data from Table 2, the algorithm described in Figure 5 computed the following water quality indicators for each source:

*q_f_* = [28.38 57.42 82.93 28.38 10.22 1.00]

The water quality indicators were used in Equation (2) from the current paper, obtaining the following priority indicator, based on water quality, for each source:

*PQ_f_* = [6.58 3.08 0.00 6.58 8.77 10.00]

The next step requires the functioning hours of each water source, as input. Those values are obtained from the Historian database, as the most recent values available for the respective tags. In our specific example, the values were the ones from Table 3.

The functioning hours from Table 3 were used in Equation (2) from [40], as *h_f_*, thus obtaining the following priority indicator, based on functioning hours, for each source:

*PH_f_* = [3.45 2.91 0.35 1.87 2.57 0.00]

The priority indicators based on water quality (*PQ_f_*) and based on functioning hours (*PH_f_*) were used, together, in Equation (1) from [40], obtaining the priority of each water source. In our specific example, the value for both α and β was set to 0.5. This means the priority indicator takes into consideration an equal contribution from the water quality and the usage degree of the sources. As a side note, when deciding a priority indicator for a source, it must be taken into account the usage degree of the source because, otherwise, overuse of the best water quality sources equipment results in more mechanical failures, which, due to the maintenance costs, make the optimizing strategy more expensive for the water company, in the long term. The values obtained for the priority of sources was:

*P_f_* = [5.01 2.99 0.17 4.23 5.67 5.00]

Performing the next step requires, as input, the minimum and maximum possible flow that each source could deliver, this information being taken from the Historian database, as the most recent values available for the respective tags. In our specific case, the values were the ones from Table 4.

The data from Table 4 was used, together with the priority indicator based on water quality (*PQ_f_*), in Equation (4) from [40], in order to compute the following optimum flow for each source:

*F_w_f_* = [7.50 8.56 8.90 10.12 9.89 15.10]

The final step in the optimizing strategy requires, as input, the total water flow that is currently requested from all sources. This value is computed inside the DWTP, by the automation which ensures the functioning of the station, but the value is set to an OPC UA tag, available for the Historian application to read. As a consequence, at the time of executing the optimization strategy, this value is taken from the Historian database, as the most recent value available for the respective tag. In our example, this value was:

*F_t_r_* = 41.14

The final step consists in dividing the total water flow requested between the sources, which, together, must provide it. This means the execution of the algorithm described in Figure 6 and Figure 7, which in the current example, provided the following optimized flows:

FAO = [7.50 0.00 0.00 8.66 9.89 15.10]

At this point, the execution of the optimizing strategy is finished. The solution provides a series of water flows that, if requested from the sources, generates the lowest possible energy consumption inside the DWTP for offering the requested flow (41.14 m^3^/h).

In the end, two additional pieces of information are displayed in the Historian GUI. The first one is the total flow offered by sources after optimization, in our case, this being equal to the requested flow (41.14 m^3^/h). However, it is possible, in certain objective circumstances, that the requested flow can’t be perfectly matched. In those special cases, the algorithm from Figure 7 was implemented to always choose to exceed the flow and not offer less than requested. If less water is offered than requested, it is possible that the water inside the distribution tank drops at such low level that no more water can be introduced in the drinking water network, although the network needs more water, this being a major incident for any DWTP. If more water is offered than requested, then the water level inside the distribution tank will grow faster, which means that the automation which operates the DWTP will reduce the requested flow faster in time, with no undesired consequences at all. Also, the second information displayed is a combination of tag name and value that the Historian alters inside the DWTP, in our example, the extract from the GUI being:

Set PF1_setpoint to 7.50
Set PF2_setpoint to 0.00
Set PF3_setpoint to 0.00
Set PF4_setpoint to 8.66
Set PF5_setpoint to 9.89
Set PF6_setpoint to 15.10

By convention, a flow set-point value of 0.00 for a water source means that the respective source will not function, i.e., it will be stopped. To sum up, the current section presented a step-by-step example of an execution of the complete automation, developed in this paper, of the energy consumption reduction strategy introduced in [40], referring actual data, outputted by the proactive Historian.

### 3.2. Test Cases for the Algorithm Which Distributes the Total Water Flow between Sources

The second objective of the test procedure, after ensuring the equations are correctly applied in the computations, was to verify if the output of the algorithm which distributes the total water flow between sources (from Figure 7) follows the guidelines drawn by the conceptual development from [40]. In an extremely simplified manner, the concept presumes that more water is requested from the highest priority sources, to the detriment of the lower priority sources, in the same time attempting to keep the requested flow for each source as close as possible to the optimum computed flow (*F_w_f_*).

In order to evaluate this secondary objective, two distinct scenarios have been considered, both involving data recorded from real world DWTPs, each one of them consisting of multiple test cases. The test procedure consisted in editing the value of the total flow requested from the sources, inside the Historian database, at the most recent record. This way, with repeated executions of the optimizing strategy, a simulation of the way in which the algorithm from Figure 7 performed, in identical conditions, for different total flow values could be assessed, independent of the real DWTP operation. This procedure enabled the execution of test cases which covered a wide range of total requested flows, in a safe environment, which could not have been executed on a real DWTP.

The first test scenario involved data recorded from a real DWTP, which had six water sources. The historical stored data analyzed by the first level algorithm covered a 4 weeks period, spanning a timeframe from 7 October 2020 to 5 November 2020. Please note that the order of sources, based on the priority indicator (*P_f_*) associated to them, as identified by the optimizing strategy, was, in this case (highest to lowest priority): 5, 1, 6, 4, 2, 3. The test cases executed in this scenario and the results that occurred have been gathered in Table 5, below, with the abbreviations explained in the Table 5 footer.

The second test scenario also involved data recorded from a real DWTP, but a different one from the DWTP used in the first scenario, this time with only four water sources. In this second scenario, the analyzed stored data covered a 2 weeks period, spanning a timeframe from 2 December 2020 to 15 December 2020. Please note that the order of sources, based on the priority indicator (*P_f_*) associated to them, as identified by the optimizing strategy, was, in this case (highest to lowest priority): 2, 4, 1, 3. In a similar approach as the one presented above, the test cases executed in this scenario and the related results compose Table 6, the abbreviations keeping the same meaning as the one detailed in Table 5 footer section.

By analyzing the test data presented in the current section, the conclusion that can be drawn is that the practical implementation of the algorithm from Figure 7 follows, indeed, the general theoretical guidance provided by the strategy proposed in [40]. Therefore, based on the results made available in the last two sections of the current paper, the complete automation of the energy consumption strategy, inside the proactive Historian, can be considered as validated.

## 4. Discussion

This section discusses and interprets the findings and results presented in the previous chapter, by also considering the perspective of previous studies. It must be highlighted that the objective of the current paper was not to prove the efficiency of the energy consumption reduction strategy, because the results had been proven in [40], where it was shown that the strategy reduced the energy consumption of a DWTP, by 9% in a model with real input data, and by 30% in a test conducted on a real process that used only a part of the algorithm.

Therefore, the main desired output of the tests executed in the current research was to prove that the developed automated method of applying the respective strategy is indeed compliant with the concept that delivered the significant energy consumption reduction results. In this regard, a series of test results were thoroughly analyzed in order to ensure all the computations based on the strategy’s equations are correct, one such test result being dissected in Section 3.1. In the same testing process, Section 3.2 presented two different scenarios, one with a DWTP containing six water sources and the other one with a DWTP containing four water sources. The results, synthesized in Table 5 and Table 6, confirmed that the automated solution is capable of choosing the water flow for each source in accordance with the guidelines of the optimization strategy.

The results of this paper must be interpreted as providing a tool, integrated inside a proactive historian type of software application, that can apply, in a completely automated manner, without requesting human assistance, an energy consumption reduction strategy inside a DWTP, in a non-invasive way regarding the station’s local automation. The complete automation from the current research means that it is now possible, after a minimum configuration required in the initial phase (setting up the objects of the process inside the Historian Process Editor and assigning meanings to the tags), to apply that strategy in practice without limitations related to human presence or knowledge. This enables the deployment of the solution on a large scale, in a fully automated long-term functioning, in the water industry, which delivers the practical benefits derived from all of the related research work to the industrial, practical, real environment.

The deployment of the now complete prototype in a real-scenario and the long-term testing will have to be allowed by the water company, and such tests are planned. Until then, the authors claim only the energy consumption reduction values obtained in [40].

Attempting a comparison between the results from this study and the results of a similar solution is not feasible at the time of writing this article because another fully automated energy consumption reduction strategy, applicable over a DWTP, could not be identified to date in either literature or practice.

The solution implemented in this paper is complementary to the local automation solution of a DWTP and acts in a non-invasive manner, the latter having priority in operation through the local open and closed-loop control strategies, basically a total control over the processes. The automatically identified priority indicators for the water wells, the process awareness, the permanent energy efficiency improvement strategy automatically obtained inside the historian considering all the constraints, the ability to automatically react over the local system and to deploy the energy efficiency strategy without human intervention, are assuring the complete automation of the historian-based energy efficiency increasing solution for a drinking water facility. The obtained priority indicators and the flow set-points for the water wells are validated regarding the energy consumption reduction strategy as in [40] in the context of a completely automated solution.

Regarding the future research directions, in a broader perspective, under this proactive Historian area, the possibilities are numerous and varied, including researches towards other kinds of optimizations inside DWTPs (e.g., substances consumptions, equipment maintenance, output water quality, etc.), researches focused on other type of facilities (e.g., wastewater treatment plants) or even studies for optimizations in different industries (e.g., energy, manufacturing, etc.). By narrowing down the perspective to the current paper’s thematic only, the obtained automated tool represents just a first version of the implementation of a conceptual development, which makes it very plausible to being susceptible to improvement. By long term observations regarding the tool’s performance in the real world, specific adjustments and enhancements can be studied and developed, in time, in order to improve the efficiency in reducing the energy consumption.

## 5. Conclusions

The current context, governed by the Industrial Internet of Things and Industry 4.0 paradigms, offers a perfect environment for researching and developing more intelligent software solutions for industrial settings. These kinds of applications of the future will be able to work autonomously in monitoring technical systems, analyzing their parameters and, ultimately, interfering in their normal operation, in order to optimize and improve various aspects, which can bring significant benefits for all involved parties.

In this general framework, and using results and developments from previous works, the current paper proposed a software tool, integrated inside a proactive Historian application, that can autonomously and automatically reduce the overall energy consumption of a DWTP. The end result of the research is a complete practical implementation of the optimizing solution, ready to be deployed into the real world.

Although a significant amount of previous work was fructified in the current research, it also brought important innovative contributions, such as: the research and development of a method of linking the results of the already available dependencies identification algorithm (from the first level of the proactive Historian reference architecture) to the quality indicators of water sources, as shown in Figure 5 and Equation (2) from current paper; the complete development of the robust algorithm that decides the exact flow each water source should provide, given a total flow that must be delivered, in order to optimize the energy consumption, in Figure 6 and Figure 7; the integration of the whole optimizing strategy inside a pre-existing Historian. All those contributions helped bridge the gaps that previously prevented the transition of the optimizing strategy towards an automated tool.

With future development directions being varied and multiple, the solution still retains a high potential to extend tangible improvements to current industrial systems. This paper’s contributions represent just a step in a long path, sustaining the tremendous effort directed towards bringing the digital power’s benefits to the many legacy systems, from different industrial branches, in order to access efficiency and performance levels, not long ago, inaccessible.

## Figures and Tables

**Table 1:** Legend of Figure 7 and the explanations of the abbreviations used in Figure 7.

Abbreviation	Explanation
WS_Used	Boolean array showing which water sources are being used and which are stopped
TFCO	Total Flow Currently Offered. This is the sum of all water flows from sources
preStartIdx	Previously Started Index. This is the index for the last water source which was started
Ft_r	The total flow requested from the water sources. This is the same as the one in Equation (5) from [40]
FAO	Flows After Optimization. This is an array containing the optimum flow for each source, computed by the optimizing strategy
N_WS	Number of Water Sources
sumAllOpt	Sum of optimum flows from all water sources
Fw_f	Array containing the optimum flow for each water source, as computed by the optimizing strategy. This is the same as the one in Equation (4) from [40]
LPRSV	Lowest Priority Running Source Value
HIGH_MAX	The highest maximum value possible
LPRSIdx	Lowest Priority Running Source Index
Pf	Array containing the priority indicator of each water source, as computed by the optimizing strategy. This is the same as the one in Equation (1) from [40]
TFCOWLPS	Total Flow Currently Offered Without Lowest Priority Source
HPIWSV	Highest Priority Idle Water Source Value
LOW_MIN	The lowest minimum value possible
HPIWSIdx	Highest Priority Idle Water Source Index
HPUWSWMFSV	Highest Priority Used Water Source Without Max Flow Set Value
HPUWSWMFSIdx	Highest Priority Used Water Source Without Max Flow Set Index
WS_MaxF	Array containing the maximum flow possible for each water source
WS_MinF	Array containing the minimum flow possible for each water source

**Table 2:** Part of the dependencies identification algorithm output which is of interest.

Water Source Number	Proportionality	Quantity
1	Not proportional	-
2	Directly proportional	29.0%
3	Directly proportional	54.6%
4	Not proportional	-
5	Inversely proportional	18.7%
6	Inversely proportional	27.4%

**Table 3:** The most recently recorded functioning hours for the water sources, at the time of optimization.

Water Source Number	Functioning Hours Value
1	3855
2	4171
3	5679
4	4781
5	4373
6	5884

**Table 4:** The most recently recorded minimum and maximum possible flow that each source can deliver, at the time of optimization.

Water Source Number	Minimum Flow (m^3^/h)	Maximum Flow (m^3^/h)
1	5.0	8.8
2	6.5	13.2
3	8.9	17.6
4	6.7	11.9
5	4.8	10.6
6	7.8	15.1

**Table 5:** The test cases executed and their results, in the first test scenario.

Test Case No.	Total Flow Requested (*F_t_r_*)	FAO 1 ^1^	FAO 2	FAO 3	FAO 4	FAO 5	FAO 6	Sum of All FAO
1	F_t_r_ < Min 5 ^2^	0	0	0	0	Min 5	0	Min 5 (>F_t_r_)
2	Min 5 < F_t_r_ < Opt 5 ^3^	0	0	0	0	F_t_r_	0	F_t_r_
3	Opt 5 < F_t_r_ < Max 5 ^4^	0	0	0	0	F_t_r_	0	F_t_r_
4	Max 5 + 1	Min 1	0	0	0	diff ^5^	0	F_t_r_
5	Max 5 < F_t_r_ < Max 5 + Opt 1	Min 1	0	0	0	diff	0	F_t_r_
6	Max 5 + Opt 1 < F_t_r_ < Max 5 + Max 1	Min 1	0	0	0	Opt 5	Min 6	>F_t_r_
7	Opt 5 + Min 1 + Min 6 < F_t_r_ < Opt 5 + Opt 1 + Min 6	diff	0	0	0	Opt 5	Min 6	F_t_r_
8	Opt 5 + Opt 1 + Min 6 + 1	diff (>Opt 1)	0	0	0	Opt 5	Min 6	F_t_r_
9	Opt 5 + Max 1 + Min 6 + 1	Opt 1	0	0	0	Opt 5	diff	F_t_r_
10	Opt 5 + Opt 1 + Opt 6	Opt 1	0	0	0	Opt 5	Opt 6	F_t_r_
11	Opt 5 + Opt 1 + Opt 6 − 1	Opt 1	0	0	0	Opt 5	diff	F_t_r_
12	Opt 5 + Opt 1 + Opt 6 + 1	Opt 1	0	0	Min 4	Opt 5	diff	F_t_r_
13	Opt 5 + Opt 1 + Opt 6 + Opt 4 + 1	Opt 1	Min 2	0	Min 4	Opt 5	Opt 6	>F_t_r_
14	Opt 5 + Opt 1 + Opt 6 + Opt 4 + Min 2 + 1	Opt 1	Min 2	0	diff	Opt 5	Opt 6	F_t_r_
15	Opt 5 + Opt 1 + Opt 6 + Max 4 + Min 2 + 1	Opt 1	Min 2	Min 3	Opt 4	Opt 5	Opt 6	>F_t_r_
16	Opt 5 + Opt 1 + Opt 6 + Opt 4 + Min 2 + Min 3 + 1	Opt 1	diff	Min 3	Opt 4	Opt 5	Opt 6	F_t_r_
17	Opt 5 + Opt 1 + Opt 6 + Opt 4 + Opt 2 + Opt 3	Opt 1	Opt 2	Opt 3	Opt 4	Opt 5	Opt 6	F_t_r_
18	Opt 5 + Opt 1 + Opt 6 + Opt 4 + Opt 2 + Opt 3 + 1	Opt 1	diff (Opt 2 + 1)	Opt 3	Opt 4	Opt 5	Opt 6	F_t_r_
19	Opt 5 + Opt 1 + Opt 6 + Opt 4 + Max 2 + Opt 3 + 1	Max 1	diff	Opt 3	Max 4	Max 5	Max 6	F_t_r_
20	Max 5 + Max 1 + Max 6 + Max 4 + Max 2 + Opt 3 + 1	Max 1	Max 2	diff	Max 4	Max 5	Max 6	F_t_r_
21	F_t_r_ > Max 5 + Max 1 + Max 6 + Max 4 + Max 2 + Max 3	Max 1	Max 2	Max 3	Max 4	Max 5	Max 6	<F_t_r_

^1^ FAO1 = Flow After Optimization of Water Source 1. ^2^ *Min 5* = Minimum Flow that Source 5 can offer. ^3^ *Opt 5* = Optimum flow for Source 5, as computed by the optimizing strategy (same as *F_w_f_*). ^4^ *Max 5* = Maximum Flow that Source 5 can offer. ^5^ diff = The difference resulted by subtracting the sum of all FAO besides current column from the value in last column of the table.

**Table 6:** The test cases executed and their results, in the second test scenario.

Test Case No.	Total Flow Requested (*F_t_r_*)	FAO 1 ^1^	FAO 2	FAO 3	FAO 4	Sum of All FAO
1	*F_t_r_* < *Min 2*	0	*Min 2*	0	0	*Min 2* (>*F_t_r_*)
2	*Min 2* < *F_t_r_* < *Opt 2*	0	*F_t_r_*	0	0	*F_t_r_*
3	*Max 2* + 1	0	diff	0	*Min 4*	*F_t_r_*
4	*Max 2* + *Min 4* + 1	*Min 1*	*Opt 2*	0	*Min 4*	>*F_t_r_*
5	*Opt 2* + *Min 4* + *Min 1* + 1	*Min 1*	*Opt 2*	0	diff	*F_t_r_*
6	*Opt 2* + *Opt 4* + *Min 1* + 1	*Min 1*	*Opt 2*	*Min 3*	*Opt 4*	>*F_t_r_*
7	*Opt 2* + *Opt 4* + *Min 1* + *Min 3* + 1	diff	*Opt 2*	*Min 3*	*Opt 4*	*F_t_r_*
8	*Opt 2* + *Opt 4* + *Opt 1* + *Min 3* + 1	diff	*Opt 2*	*Opt 3* (=*Min 3*)	*Opt 4*	*F_t_r_*
9	*Opt 2* + *Opt 4* + *Opt 1* + *Opt 3* < *F_t_r_* < *Max 2* + *Max 4* + *Max 1* + *Max 3*	diff	*Opt 2*	*Opt 3*	*Opt 4*	*F_t_r_*
10	*F_t_r_* > *Max 2* + *Max 4* + *Max 1* + *Max 3*	*Max 1*	*Max 2*	*Max 3*	*Max 4*	<*F_t_r_*
11	*Max 2* + *Opt 4* + *Opt 1* + *Opt 3* + 1	diff	*Opt 2* (=*Max 2*)	*Opt 3* (=*Min 3*)	*Opt 4*	*F_t_r_*

^1^ FAO1 = Flow After Optimization of Water Source 1.

## Data Availability

Not applicable.
